# Basal Ganglia Compensatory White Matter Changes on DTI in Alzheimer’s Disease

**DOI:** 10.3390/cells12091220

**Published:** 2023-04-23

**Authors:** Zdeněk Wurst, Barbora Birčák Kuchtová, Jan Křemen, Anastasiya Lahutsina, Ibrahim Ibrahim, Jaroslav Tintěra, Aleš Bartoš, Marek Brabec, Tanya Rai, Petr Zach, Vladimír Musil, Nicoletta Olympiou, Jana Mrzílková

**Affiliations:** 1 Department of Anatomy, Third Faculty of Medicine, Charles University, Ruska 87, 100 00 Prague, Czech Republic; 2 Klinik für Neurologie, Universitätsklinikum Schleswig-Holstein Campus Lübeck, Ratzeburger Allee 160, 23562 Lübeck, Germany; 3 Department of Radiodiagnostic and Interventional Radiology, Institute for Clinical and Experimental Medicine, Videnska 1958/9, 140 21 Prague, Czech Republic; 4 Department of Neurology, Third Faculty of Medicine, University Hospital Kralovske Vinohrady, Charles University, Ruska 87, 100 00 Prague, Czech Republic; ales.bartos@lf3.cuni.cz; 5 Department of Statistical Modeling, Institute of Computer Science, Academy of Sciences of the Czech Republic, Pod Vodarenskou vezi 271/2, 182 07 Prague, Czech Republic; 6 Centre of Scientific Information, Third Faculty of Medicine, Charles University, Ruska 87, 100 00 Prague, Czech Republic

**Keywords:** DTI, Alzheimer’s disease, basal ganglia, white matter, compensatory changes

## Abstract

The volume reduction of the gray matter structures in patients with Alzheimer’s disease is often accompanied by an asymmetric increase in the number of white matter fibers located close to these structures. The present study aims to investigate the white matter structure changes in the motor basal ganglia in Alzheimer’s disease patients compared to healthy controls using diffusion tensor imaging. The amounts of tracts, tract length, tract volume, quantitative anisotropy, and general fractional anisotropy were measured in ten patients with Alzheimer’s disease and ten healthy controls. A significant decrease in the number of tracts and general fractional anisotropy was found in patients with Alzheimer’s disease compared to controls in the right caudate nucleus, while an increase was found in the left and the right putamen. Further, a significant decrease in the structural volume of the left and the right putamen was observed. An increase in the white matter diffusion tensor imaging parameters in patients with Alzheimer’s disease was observed only in the putamen bilaterally. The right caudate showed a decrease in both the diffusion tensor imaging parameters and the volume in Alzheimer’s disease patients. The right pallidum showed an increase in the diffusion tensor imaging parameters but a decrease in volume in Alzheimer’s disease patients.

## 1. Introduction

Structural changes of the basal ganglia (BG) are typically the domain of the neurodegenerative diseases, such as Parkinson’s disease (PD). Conversely, there are not many studies of the white matter (WM) changes around the principal BG (e.g., putamen, caudate and pallidum) in Alzheimer’s disease (AD). In a three-year longitudinal study of AD, unilateral atrophy in the right caudate nucleus and bilateral atrophy in the putamen was reported [1]. However, after the progression of AD over time this atrophy was also found in the left caudate nucleus [1]. Besides the morphological changes, the differences in the BG perfusion were also found in AD. For example, hyperperfusion in the right putamen and in the head of the right caudate nucleus was observed in magnetic resonance imaging (MRI) in AD [2]. Thus, it is plausible that changes in the perfusion of the BG may lead with time to hypo- or hypertrophy of the WM located nearby [3,4]. Even though these changes in the perfusion may be typical for vascular dementia, hyper-perfusion could lead to the opposite effect, such as an increase in the structural and functional parameters. In the carriers of the apolipoprotein E (APOE) epsilon 4 allele with AD, the regional blood supply to many areas of the cerebral cortex and subcortical structures was significantly and asymmetrically reduced. [5].

Both diffusion-weighted imaging (DWI) and diffusion tensor imaging (DTI) are promising methods that can be used to assess the microstructure of axonal bundles and depict the axonal integrity of both normal and pathological brain tracts based on the diffusion properties of the brain tissue [6].

In our previous study [7], we found a significant reduction in the diffusion tensor imaging parameters in the fornix of AD patients compared to controls, likely due to neuronal degeneration and white matter loss. However, we simultaneously observed a surprising increase in values of tractographic parameters in the subcallosal area and the paraterminal gyrus in the patients with AD compared to the control group. Our explanation was that the patients with AD compensate for the loss of the ability to consolidate memory by redirecting and utilizing other structures, such as the subcallosal area and the paraterminal gyrus, especially if the fornix fibers are affected at the same time.

Given our previous data on DTI-based tractography demonstrating asymmetrical compensatory changes in the white matter structure of the subcallosal area and the paraterminal gyrus in AD patients, the present research aimed to determine whether there are compensatory changes in the findings related to the white matter diffusion tensor imaging data in the motor basal ganglia of the AD patients compared to the healthy controls. Our hypothesis was that the AD patients would show an increase in the DTI parameters (NT and/or QA) in some but not all of the BG compared to the healthy controls.

## 2. Materials and Methods

### 2.1. Subjects/Participants

In this study, we recruited 10 patients with a confirmed AD diagnosis and 10 healthy controls (Table 1). MRI and mini-mental state examination (MMSE) tests were performed on all subjects at the Alzheimer’s Disease Center, Department of Neurology, Third Faculty of Medicine, Charles University, Prague, Czech Republic. For the purpose of the study, we used two groups of participants: (1) patients with mild cognitive impairment and dementia caused by AD according to NIA-AA criteria [8,9] and (2) cognitively normal older adults. At the beginning of the study, there was a separate third MCI patient group, which was classified as the AD group later since all the MCI group participants got diagnosed with AD by the end of the study. The AD diagnosis was made by an experienced neurologist through a thorough neurological and neuropsychological examination, functional assessments, blood work-up, brain MRI, single photon emission computed tomography (SPECT), and measurements of the total and phosphorylated tau proteins as well as β-amyloid peptides in the cerebrospinal fluid upon the patients’ consent to a lumbar puncture [10]. Most patients with the diagnosis were followed up for several years before they showed a cognitive and functional decline. Adults (controls) with normal cognitive abilities were recruited for the study at the University of the Third Age (adult education courses) of the Third Faculty of Medicine, Charles University, Czech Republic. These had normal MMSE scores (given recent Czech norms and limits) for mild AD [11]. In the control group, only those who were over 70 years of age were selected in order to stay consistent with the age of the AD patients. For a more detailed description, see our previous article [6]. The research was approved by the Ethics Committee of the Prague Psychiatric Center/National Institute of Mental Health and the Human Ethics Committee of the Third Faculty of Medicine, Charles University, Prague, Czech Republic, (Protocol No. 2016/3), and informed consent was obtained for all patients according to the Declaration of Helsinki.

### 2.2. MRI Data Acquisition

All the recruited subjects in the present study were scanned on a 3T MRI scanner (Siemens Magnetom Trio, Erlangen, Germany) using a 24-channel head coil (adaptive coil combine mode was used) according to the following procedure:

(1) T1-weighted 3D MPRAGE had the following parameters: voxel size of 0.85 × 0.85 × 0.85 mm^3^, 192 sagittal slices, TE of 4.73 ms, TR of 2000 ms, flip angle of 10°, FOV of 326 mm, and TA:10:42 min.
(2) 3D T2-weighted FLAIR had the following parameters: voxel size of 1 × 1 × 1 mm^3^, 176 sagittal slices, TE of 422 ms, TR of 6000 ms, FOV of 256 mm, and TA: 6:38.
(3) Diffusion-weighted images using SE EPI sequence had the parameters: voxel size of 2 × 2 × 2 mm^3^, TR of 6000 ms, TE of 93 ms, 44 axial slices, three averages, FOV of 256 mm, number of diffusion directions 20, and two b values: 0, 1000 s/mm^2^, TA: 6:38 min.

### 2.3. DTI Analysis

The DTI data were adjusted for distortion and countercurrents using the FSL Studio program (www.fmrib.ox.ac.uk/fsl/index.html, accessed on 8 March 2023). For correction of the head motion and eddy current distortion, the Eddy program within FSL (version 6.0.1) was used [12]. The DTI image set having b = 0 EPI was co-registered to T1-weighted 3D MPRAGE to obtain a co-registration matrix; this was further used for other EPI diffusion images. FLIRT (FMRIB’s Linear Image Registration Tool), which is a fully automated robust tool for affine (linear) inter- and intra-modal registration, was used [13]. A Tri-Linear interpolation method in the final (reslice) transformation was performed using FLIRT/Advanced Options.

The potential influence of various factors leading to bias (e.g., use of only one MRI scanner or system errors) or distortion in the results is discussed in our previous article [7].

### 2.4. Anatomical Considerations

All the analyzed BG structures were manually delineated by two experienced anatomists to precisely differentiate between the GM and the WM structures (the internal and the external capsule). All the DTI parameters were measured on the WM surrounding the BG, although the BG themselves were used as a landmark for the anatomical orientation in the WM (see the supplementary material videos: R caudate ROI, R pallidum ROI, and L putamen ROI).

### 2.5. DTI Data Reconstruction

The diffusion-weighted imaging (DWI) data were first corrected for distortions and countercurrent effects using the FSL; then, the data were evaluated in the DSI studio using the generalized q-sampling imaging (GQI) algorithm with the Q-space diffeomorphic reconstruction (QSDR), thus reconstructing the data in the MNI space. QSDR is a model-free method that calculates an approximate density distribution of diffusing water in a standard space to preserve the orientation of fibers so that they can be traced [14]. The diffusion gradient table (see the Supplement Table S1) was rotated for each data unit according to the co-registration matrix before proceeding with the QSDR.

### 2.6. Tractography

The fiber tracking was performed using the following tracking parameters: the anisotropy (nQA) threshold was set at 0.05, the angular threshold was 60°, and the step size was 1 mm. The tracts that were less than 60 mm in length were not counted. A total of 1,000,000 seeds were placed. The obtained values were used for further statistical procedures.

### 2.7. Measured Parameters

The DTI tractography resulted in the following values and parameters: the number of tracts (NT), tract length (TL), tract volume (TV), quantitative anisotropy (QA) as a marker of the tract directionality, and general fractional anisotropy (GFA) as a marker of the tract connectivity (Table 2). These parameters were analyzed further.

### 2.8. FreeSurfer Volume Analysis

The MRI images were processed with the latest version of the freely available reconstruction software FreeSurfer (FS) (version: v6.0; http://surfer.nmr.mgh.harvard.edu (accessed on 8 March 2023)). This software creates a virtual 3D reconstruction of the brain structures from magnetic resonance images [15]. The DICOM MRI images from the AD patients and controls were transferred to the FS software environment in a standard way. For the purpose of the study, the values of the basal ganglia volumes, and the total brain volume (white and grey matter) were used. After processing, all the data were stored in an Excel spreadsheet for further statistical evaluation.

### 2.9. Statistical Analysis

**Figure 1:**
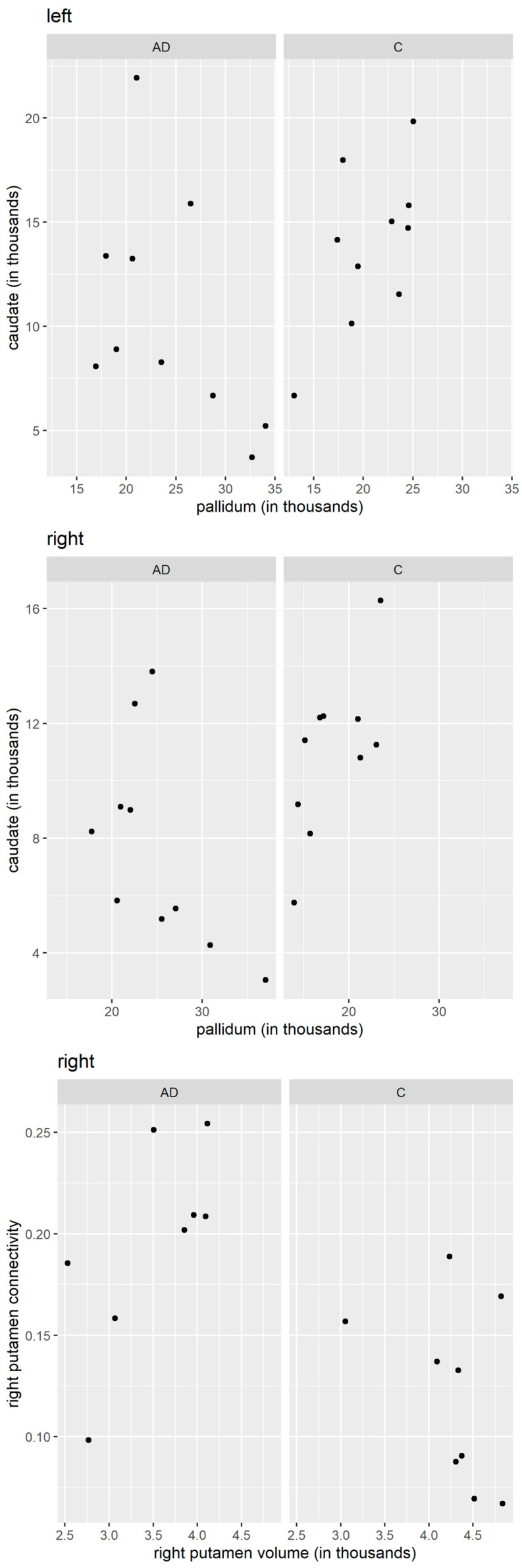
Comparison of the number of tracts, connectivity, and volume in the caudate, pallidum, and putamen (Pearson correlation coefficients). Upper panels: left pallidum vs. left caudate, number of tracts. Middle panels: right pallidum vs. right caudate, number of tracts. Bottom panels: right putamen volume vs. right putamen connectivity. AD—Alzheimer’s disease patients; C—controls. Values on the x- and y-axis are reported in absolute units.

The statistical analysis was performed using the STATISTICA 13 software and the R statistical computing environment [16]. The two-way ANOVA with repeated measures was applied for the analysis of the two independent groups (the AD patients and controls) and the two dependent variables (the left and the right side). The Wilks lambda test was used to evaluate the differences between the AD and control groups with the left and right sides as variables for both the DTI analysis and the FreeSurfer volumetric analysis (Table 2 and Table 3). Pearson correlation coefficients were calculated to assess the relationship between the measures taken for the same person at different locations (Figure 1). Subsequently, we tested the differences in these correlations computed in the AD and control groups separately, using the Fisher z-transform [17] and the two-sided test (for which we cite *p*-values). In particular, we tested the AD versus control difference in correlations for both the number of tracts and the connectivity characteristics, comparing—(i) the laterality (correlating the left and the right value of the same patient) of the putamen, pallidum, and the caudate, (ii) the structure (correlating the putamen, pallidum, and the caudate, separately for the left and right hemisphere), and then (iii) the structure segmentation volume (correlating the brain segmentation volumes in the putamen, pallidum, and the caudate, separately for the left and right hemisphere), plus (iv) the number of tracts/connectivity related to the volume of the putamen, pallidum, and the caudate. We acknowledge that different characteristics (like the number of tracts on the left and on the left) might be, to some extent, correlated.

The volume datasets from the FS program were extracted, converted into Excel spreadsheets, and subsequently processed in the STATISTICA software.

## 3. Results

The age, education, sex, and MMSE score comparison between the AD patients and the healthy controls are shown in Table 1. The samples of the 3D rotatory videos of the caudate, pallidum, and putamen DTIs, as well as their ROIs, are included in the supplementary material (Caudate AD, Caudate ctrl, Pallidum AD, Pallidum ctrl, Putamen AD, Putamen ctrl; R caudate ROI, R pallidum ROI, L putamen ROI).

### 3.1. DTI Analysis

Differences between the AD patients and the healthy controls were observed only in the NT and the normalized quantitative anisotropy (nQA). There were no significant differences in the TL, TV, and QA, as reported in Table 2.

### 3.2. Number of Tracts (NT)

**Figure 2:**
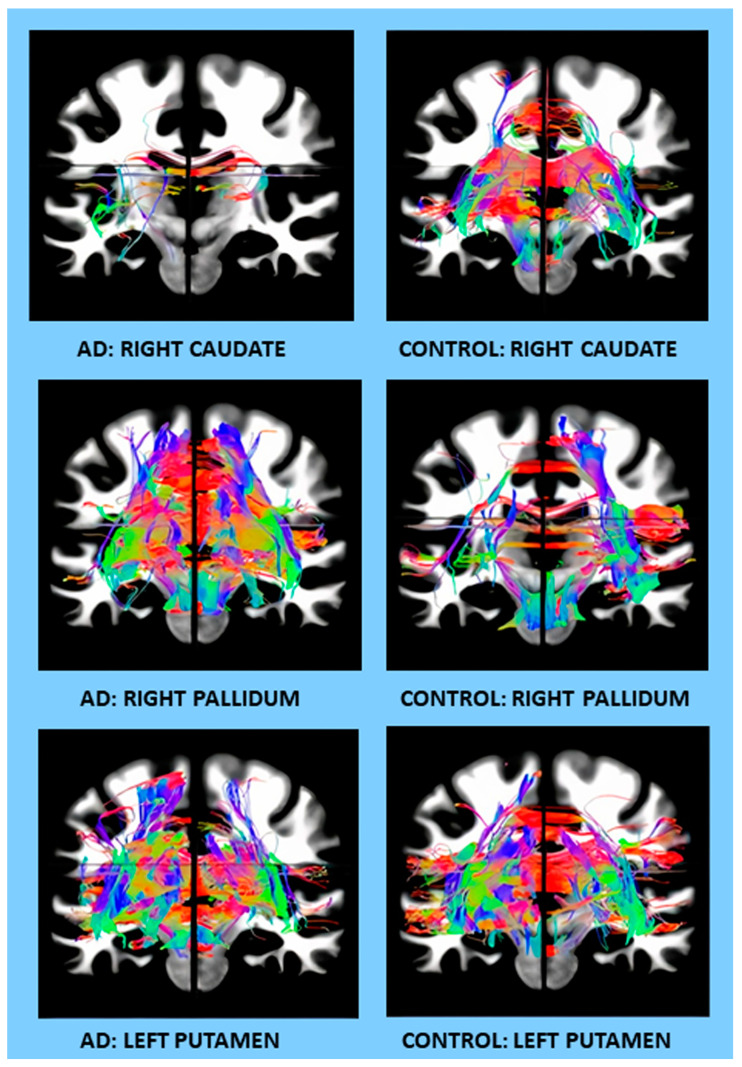
Examples of DTI changes in AD patients compared to controls on coronal sections for different brain regions (the right caudate, right pallidum, and the left putamen).

Compared to the controls, the patients with AD (AD ± SD/ctrl ± SD) showed decreased NT in the right caudate (7667 ± 3557/10,945 ± 2816) and increased in the right pallidum (24,882 ± 5633/18,202 ± 3649), the right putamen (38,715 ± 9724/27,172 ± 5618, and the left putamen (42,603 ± 9387/35,368 ± 4250) (Figure 2, Table 2).

### 3.3. Normalized Quantitative Anisotropy (nQA)

Patients, compared to controls, showed higher nQA values in the right pallidum (0.21 ± 0.05/0.15 ± 0.06), left pallidum (0.21 ± 0.05/0.15 ± 0.06), right putamen (0.19 ± 0.05/0.13 ± 0.05), and the left putamen (0.2 ± 0.05/0.15 ± 0.06) (Table 2).

### 3.4. FreeSurfer Volume Analysis

The differences between the patients with AD and the controls were observed in the volume of the left and right putamen and in the nucleus accumbens area. On the other hand, no significant differences were observed in the left and right caudate nucleus and also in the brain segmentation volume. Further, a volume decrease in the right and left putamen was found in the AD patients in comparison to controls (respectively, 3487.3 µm^3^ vs. 4283.9 µm^3^ and 3656.5 µm^3^ vs. 4302.2 µm^3^), as reported in Table 3.

### 3.5. Pearson Correlation Coefficients

Significant changes in the correlations of several characteristics were observed between the patients with AD and the controls. In particular, the correlation between the number of tracts in the left pallidum and the number of tracts in the left caudate was 0.609 for the controls and −0.493 for the AD patients. The difference was highly significant, with the *p*-value = 0.019. The correlation between the number of tracts in the right pallidum and the number of tracts in the right caudate was 0.691 for the controls and −0.566 for the AD patients; hence, their difference was highly significant, *p*-value = 0.005. The correlation between the right putamen connectivity and the right putamen volume was −0.367 for the controls and 0.678 for the AD patients; hence, their difference was significant, with the *p*-value = 0.04. The qualitative differences in correlation patterns between the AD patients and the controls are shown graphically in Figure 1.

## 4. Discussion

DTI is challenging, and the fiber tract reconstruction depends on the quality of the diffusion data. The theoretical basics and a number of factors influencing the reconstruction results are covered in the literature [18,19].

For example, the gradient field inhomogeneity causes artifacts that affect the results of the reconstructed fibers. The gradient field can be efficiently mapped using the b-matrix spatial distribution in DTI (BSD-DTI) technique to correct the magnitude and the direction of the diffusion gradient [20].

In our study, the nQA parameter has been used for the fiber tracking (FT) instead of the FA due to the fact that QA-aided tractography has reached a better resolution and is less sensitive to partial volume effects of the crossing fibers than the tractography based on the FA [21]. FA is defined for all the fiber populations within a voxel and suffers from the partial volume effect.

Therefore, the data were reconstructed using the DSI studio with the GQI method. GQI is a free model that can be applied to any diffusion scheme that provides a quantitative anisotropy (QA) parameter, which is based on the spin distribution function (SDF) of diffusing water at different orientations. QA measures the spin density of anisotropy along a fiber pathway for each fiber population and contributes to more reliable tractography. QA can be normalized (nQA), which stabilizes the proton density across subjects [22].

An increase in values of the quantitative DTI parameters in the WM of the AD patients was observed only in the left and the right putamen, while their volumes were reduced compared to the controls. The right caudate showed, as expected, a decrease in both the DTI parameters and the volume in the AD patients compared to the controls. The right pallidum showed, similarly to the putamen, an increase in the DTI parameters but a decrease in volume in the AD patients compared to the controls.

An increase in the values of the quantitative DTI parameters of the WM observed in AD patients suggests the plasticity of specific tracts. The question is whether the whole process should be labeled as degeneration. Deposition of the amyloid plaques and deposits is typically present at the inferior part of the temporal lobe and the posterior cingulum [10,23]. These are also anatomical targets of the projections that undergo the WM hypertrophy or increase in the tract fibers on the DTI.

Why is there a decrease in the number of tracts in the caudate nucleus but an increase in the pallidum and the putamen? When considering the loops of the BG circuits, the motor loop skips the caudate nucleus but not the pallidum and the putamen. On the contrary, the executive/associative loop of the BG skips the putamen but deploys the caudate nucleus [24]. Since the motor skills are not affected in the early and mid-stages of AD, while cognition and memory decline, the observed compensatory WM hypertrophy in the putamen and the pallidum does not seem to be effective even though it is present. Since the caudate nucleus inhibits the pallidum, the increase in the WM of the pallidum could be due to its spontaneous activation after the caudate atrophies.

Given that the caudate is evolutionarily older compared to the relatively younger putamen [25], we hypothesize that the caudate could be the first to suffer the loss of structure and function. Thus, the putamen may not undergo neurodegeneration so easily; it could stay intact for longer and may, to some extent, substitute for the loss of function of the caudate. However, the observed caudate/putamen volume ratio in early caudate dysfunction in PD patients suggests this is not the case [26]. Although the WM compensatory changes in AD on the DTI were not described frequently, they were observed in PD [27], Tourette’s syndrome [28], schizophrenia, and bipolar disorder [29,30]. Interestingly, increased connectivity in the right caudate nucleus was observed in cognitively normal PD patients [31].

Recently, we proposed that an increase in values of the quantitative DTI parameters of the WM in the subcallosal area and the paraterminal gyrus is an aftermath of the hippocampal atrophy [7,32]. We now propose another structural/functional compensatory mechanism for hippocampal atrophy in AD in terms of the BG white matter volume increase. The reason for this compensatory hypertrophy could be their participation in the association loop of the BG circuit (association cortex—BG—thalamus—cortex). This circuit is responsible not only for motor skills but also for memory formation (emotional memory and positive reward reaction, episodic memory, and association cortices bound to memory formation).

In AD, attention has been paid to the brain areas with clinically proven morphological atrophy (the hippocampus, various cortical areas, the brain stem, and others). Recently, there were attempts to include clinical diagnostics and atrophy of neuroanatomical heterogeneous areas, i.e., basal forebrain cholinergic system [33].

We suggest another option: what if there are numerous compensatory shifts in motor/association/sensory and other brain structures, including the tracts in AD patients, detectable on the DTI (such as NT, TL, TV, QA, and GFA) that manifests by default when the atrophied primary memory circuits fail to work properly?

Limitations of the study: A small sample size (10 subjects) of our study represents some limitations; the observed asymmetric changes may be the result of a small sample, and further study with more patients is needed to confirm our conclusions. White matter changes around the basal ganglia were not specifically parcellated into the afferents or the efferents, nor were they classified into any kind of intrinsic or extrinsic projections in regards to the cortex, the brain stem, or the diencephalon. This way, it was compared only to the sum of three-directional projections between the AD patients and the controls. For future research it would be good to compare the DTI-based tractography of separate tracts and pathways of the BG in AD patients and controls, the cortical ones in particular.

## 5. Conclusions

Our data show there is an asymmetrical increase in the DTI parameters in patients with AD, which is consistent with our hypothesis stating that the same pattern may appear in other brain areas as well (which has not been proven yet). More specifically, a decrease in the volume of the left and the right putamen in the AD patients compared to controls was expected when measured by the FS. Interestingly, there was an increase in the NT in their proximity. If this was the effect of the compensatory changes (i.e., reduced volume of the structure and an increase in the amount of the WM fibers around it), then it remains unclear why the pallidum or caudate would not show a similar compensatory effect as well. Moreover, the timing of these changes remains unclear. Do they arrive prior to the decrease in the volume of the putamen and the increase in the white matter NT follows, or is it the other way around? Or rather, do all the changes occur relatively simultaneously?

## Figures and Tables

**Table 1:** Characteristics of AD and control group.

	AD Group	Control Group	*p* Values
Numbers of participants	10	10	
Age at scan (years)	70.1 ± 6.5	67.6 ± 4.2	n.s
Education (years)	13 ± 1	14 ± 6	n.s
Male/female sex	6/10	5/10	n.s
MMSE score (0–30 pts.)	21 ± 3	29 ± 8	*p* < 0.001

Data are expressed as mean ± standard deviation. MMSE—the Mini-Mental State Examination, n.s.—not significant.

**Table 2:** Overview of the DTI parameters in patients with Alzheimer’s disease and controls. NT = the number of tracts, TL = tract length, TV = tract volume, QA = quantitative anisotropy, nQA = normalized quantitative anisotropy, GFA = generalized fractional anisotropy, unit = stands for absolute numbers, AD = Alzheimer‘s disease patients, ctrl = healthy control group. The data are reported as mean values ± standard deviation (SD); * *p* ≤ 0.01, ** *p* ≤ 0.001.

	NT (Unit)	nQA (Unit)	TV (mm^3^)	GFA (Unit)	QA (Unit)	TL (mm)
right caudate ctrl	10,945 ± 2816 *	0.13 ± 0.05	46,594 ± 16,341	0.1 ± 0.004	0.6 ± 0.13	71 ± 11.1
right caudate AD	7667 ± 3557 *	0.17 ± 0.05	39,382 ± 18,303	0.09 ± 0.006	0.62 ± 0.13	68.6 ± 15
left caudate ctrl	13,873 ± 3813	0.13 ± 0.05	55,388 ± 16,471	0.1 ± 0.005	0.61 ± 0.13	77.6 ± 8.1
left caudate AD	10,527 ± 5558	0.18 ± 0.06	46,135 ± 21,453	0.1 ± 0.01	0.66 ± 0.15	74.6 ± 16.2
right pallidum ctrl	18,202 ± 3649 **	0.15 ± 0.06 *	87,140 ± 30,651	0.11 ± 0.003	0.68 ± 0.1	110 ± 16.8
right pallidum AD	24,882 ± 5633 **	0.21 ± 0.05 *	107,239 ± 23,390	0.11 ± 0.003	0.74 ± 0.14	115.8 ± 16
left pallidum ctrl	20,728 ± 4002	0.15 ± 0.06 *	92,296 ± 23,687	0.11 ± 0.005	0.7 ± 0.09	116.3 ± 13.4
left pallidum AD	24,105 ± 6108	0.21 ± 0.05 *	102,759 ± 23,521	0.11 ± 0.005	0.74 ± 0.15	117.8 ± 19.3
right putamen ctrl	27,172 ± 5618 **	0.13 ± 0.05 *	102,896 ± 35,726	0.1 ± 0.004	0.62 ± 0.12	98.2 ± 15.9
right putamen AD	38,715 ± 9724 **	0.19 ± 0.05 *	127,691 ± 30,643	0.1 ± 0.005	0.7 ± 0.12	104 ± 9.7
left putamen ctrl	35,368 ± 4250 *	0.15 ± 0.06 *	115,491 ± 23,729	0.1 ± 0.004	0.68 ± 0.11	104.6 ± 11.3
left putamen AD	42,603 ± 9387 *	0.2 ± 0.05 *	128,184 ± 29,395	0.1 ± 0.007	0.74 ± 0.16	107.8 ± 15

**Table 3:** The difference in the basal ganglia structures volumes and the total brain volume in Alzheimer’s disease patients and controls as estimated by FreeSurfer. The values are reported in µm^3^, n.s. = not significant.

Structure	AD Patients	Controls	*p*-Value
left caudate	3016.7	3304.8	n.s.
left putamen	3656.5	4302.2	*p* = 0.01
left pallidum	1843.1	1747.9	n.s.
right caudate	3043.4	3321.4	n.s.
right putamen	3487.3	4283.9	*p* = 0.01
right pallidum	1897.5	1813.8	n.s.
brain segmentation volume	10.1 × 10^5^	10.37 × 10^5^	n.s.

## Data Availability

The data presented in this study are available on request from the corresponding author. The data are not publicly available due to ethical reasons.

## Supplementary Materials

The following supporting information can be downloaded at: https://www.mdpi.com/article/10.3390/cells12091220/s1; Supplementary material contains nine example videos (AVI format) of DTI tractography and region of interest (ROI) delineation of caudate, pallidum, and putamen. Example video files: Video S1: Caudate AD; Video S2: Caudate ctrl; Video S3: Pallidum AD; Video S4: Pallidum ctrl; Video S5: Putamen AD; Video S6: Putamen ctrl; Video S7: R caudate ROI; Video S8: R pallidum ROI; Video S9: L putamen ROI. Table S1: The diffusion gradient table.

## Abbreviations

AD—Alzheimer’s disease; APOE—apolipoprotein E; BFCS—basal forebrain cholinergic system; BG—basal ganglia; DTI—diffusion tensor imaging; DWI—diffusion-weighted imaging; FS—FreeSurfer; GFA—general fractional anisotropy; MMSE—mini-mental state examination test; MRI—magnetic resonance imaging; nQA—normalized quantitative anisotropy; NT—number of tracts; PD—Parkinson’s disease; QA—quantitative anisotropy; QSDR—Q-space diffeomorphic reconstruction; SPECT—single photon emission computed tomography; TL—tract length; TV—tract volume; WM—white matter.
