# Supplementary material for: Basal Ganglia Compensatory White Matter Changes on DTI in Alzheimer’s Disease
Source: Cells. 2023 Apr 23;12(9):1220. doi: 10.3390/cells12091220 (PMC10177535; doi:10.3390/cells12091220)
Supplement: Supplementary file 1 [file cells-12-01220-s001.zip › Table.pdf]

Table S1: – The diffusion gradient table

b-value	bx	by	bz
1000	0.999977	0.00497849	-0.00458171
1000	0.00099493	-0.936941	-0.349487
1000	-0.0310767	-0.53884	-0.841834
1000	0.854539	-0.518459	-0.0310261
1000	0.835692	-0.131788	-0.533151
1000	-0.834089	-0.454864	0.31208
1000	-0.85618	-0.413282	-0.310086
1000	0.823194	-0.206564	0.52885
1000	0.549805	-0.657055	0.515745
1000	0.465895	-0.884621	-0.0196771
1000	0.515064	-0.660448	-0.546367
1000	0.392191	-0.21591	-0.894186
1000	-0.477904	-0.316602	0.819372
1000	-0.391041	-0.75641	0.524338
1000	-0.513632	-0.857803	-0.0188663
1000	-0.467154	-0.680681	-0.564306
1000	-0.551367	-0.147572	-0.821107
1000	-0.109788	0.0967661	-0.989233
1000	0.109823	-0.591188	0.799021
1000	0.0308357	-0.961942	0.271508
1000	0.999977	0.00497849	-0.00458171
1000	0.00099493	-0.936941	-0.349487
1000	-0.0310767	-0.53884	-0.841834
1000	0.854539	-0.518459	-0.0310261
1000	0.835692	-0.131788	-0.533151
1000	-0.834089	-0.454864	0.31208
1000	-0.85618	-0.413282	-0.310086
1000	0.823194	-0.206564	0.52885
1000	0.549805	-0.657055	0.515745
1000	0.465895	-0.884621	-0.0196771
1000	0.515064	-0.660448	-0.546367
1000	0.392191	-0.21591	-0.894186
1000	-0.477904	-0.316602	0.819372
1000	-0.391041	-0.75641	0.524338
1000	-0.513632	-0.857803	-0.0188663
1000	-0.467154	-0.680681	-0.564306
1000	-0.551367	-0.147572	-0.821107
1000	-0.109788	0.0967661	-0.989233
1000	0.109823	-0.591188	0.799021

1000	0.0308357	-0.961942	0.271508
1000	0.999977	0.00497849	-0.00458171
1000	0.00099493	-0.936941	-0.349487
1000	-0.0310767	-0.53884	-0.841834
1000	0.854539	-0.518459	-0.0310261
1000	0.835692	-0.131788	-0.533151
1000	-0.834089	-0.454864	0.31208
1000	-0.85618	-0.413282	-0.310086
1000	0.823194	-0.206564	0.52885
1000	0.549805	-0.657055	0.515745
1000	0.465895	-0.884621	-0.0196771
1000	0.515064	-0.660448	-0.546367
1000	0.392191	-0.21591	-0.894186
1000	-0.477904	-0.316602	0.819372
1000	-0.391041	-0.75641	0.524338
1000	-0.513632	-0.857803	-0.0188663
1000	-0.467154	-0.680681	-0.564306
1000	-0.551367	-0.147572	-0.821107
1000	-0.109788	0.0967661	-0.989233
1000	0.109823	-0.591188	0.799021
1000	0.0308357	-0.961942	0.271508
